# Case Report: Recurrent supraglottitis in a patient with metastatic melanoma treated with ipilimumab and nivolumab

**DOI:** 10.3389/fonc.2025.1615835

**Published:** 2025-08-27

**Authors:** Marisa Palmeri, Sarah A. Weiss, Phillip Purnell

**Affiliations:** ^1^ Rutgers Robert Wood Johnson School of Medicine, New Brunswick, NJ, United States; ^2^ Medical Oncology, Rutgers Cancer Institute, New Brunswick, NJ, United States; ^3^ Department of Otolaryngology, Robert Wood Johnson University Hospital, New Brunswick, NJ, United States

**Keywords:** immunotherapy, immune checkpoints inhibitors, immune-related adverse events, airway disease, supraglottitis

## Abstract

**Background:**

Immune checkpoint inhibitors (ICIs) are a standard of care for advanced melanoma but have the potential to cause immune-related adverse events (irAEs), which can vary widely in character and severity. Immune-related airway disease, such as supraglottitis, is a lesser-known and likely underreported irAE. More attention is needed to recognize this toxicity as delay has the potential to lead to airway compromise. Furthermore, the decision on whether to retreat after this toxicity poses a clinical challenge.

**Case presentation:**

A 73-year-old woman with metastatic mucosal melanoma on ipilimumab/nivolumab presented with cough, dysphagia, and globus sensation. Video laryngoscopic evaluation diagnosed supraglottic edema, which was responsive to steroids; however, it recurred twice after ICI rechallenge with nivolumab monotherapy.

**Conclusions:**

This case identifies supraglottitis as a rare irAE that requires prompt recognition to avoid airway obstruction. Close multidisciplinary collaboration with an otolaryngologist is imperative for early recognition and co-management. Reinitiation of immunotherapy after this irAE requires a careful assessment because underlying inflammation may persist and supraglottitis may recur. Further study should focus on the prevention of and prophylaxis for recurrent irAEs, particularly in patients whose disease is responding to ICI and may benefit from further treatment.

## Introduction

Immune checkpoint inhibitors (ICIs) are a mainstay of treatment for several advanced cancers. In melanoma, anti-PD-1-based ICI regimens are often used as first-line therapy in the neoadjuvant, adjuvant, and metastatic settings. While combination immune checkpoint inhibition with ipilimumab and nivolumab has yielded impressive overall survival rates for advanced melanoma, the incidence of grade 3–4 immune-related adverse events (irAEs) is high (1, 2). These events may require immunosuppression, disrupt treatment, and adversely affect quality of life. The most common irAEs such as rash, endocrinopathies, arthritis, colitis, and hepatitis have been well-characterized clinically, but any organ system can be affected. Currently, no validated tools exist to predict irAE development, and prophylactic strategies are lacking. Moreover, the decision to resume ICI therapy after an irAE remains clinically complex.

Immune-related airway disease is a lesser-known and potentially underreported irAE (3, 4). Here, we describe a rare case of a patient with stage IV mucosal melanoma treated with ipilimumab and nivolumab who developed supraglottitis. Supraglottitis is characterized by inflammation of the epiglottis or other supraglottic structures, such as the arytenoids and aryepiglottic folds. To our knowledge, ICI-induced supraglottitis has only been reported in two other cases in the literature (3). A summary of published cases of ICI-induced upper airway disease is summarized in **Table 1**. We aim to raise awareness of upper airway irAEs and emphasize the importance of timely recognition to prevent life-threatening complications.

**Table 1:** Summary of published cases of ICI-induced supraglottitis.

Citation	Patient demographics	Primary cancer type	ICI agents	Time to symptom onset	Symptoms	Treatment	Treatment outcome	Retreatment with ICI (Y/N)
Gascon et al., 2024 (3)	62yo M	Melanoma	Nivolumab + relatlimab	5 months	Dysphonia, dysphagia	High-dose IV corticosteroids and IV antibiotics	Supraglottitis recurred after completion of an initial steroid taper and required a second course of steroids	N
Gascon et al., 2024 (3)	76yo M	Bladder	Pembrolizumab	4 months	Hoarseness, throat discomfort	Corticosteroids, IV antibiotics, and antifungal	Clinical improvement although had persistent supraglottic swelling without airway obstruction	Y; was maintained on daily low-dose steroids for secondary prophylaxis

## Case description

**Figure 1:**
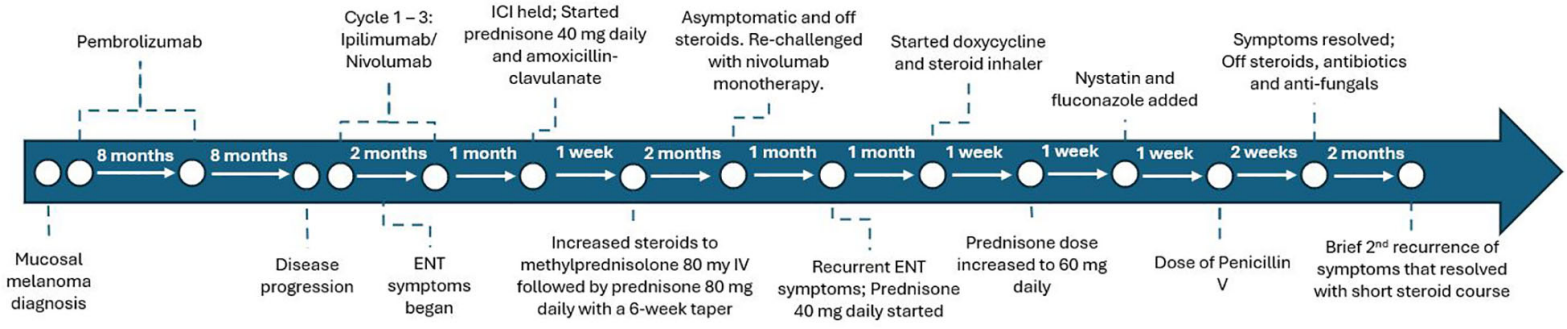
Timeline of the patient’s cancer therapy and adverse event management.

A 73-year-old woman with psoriasis and BRAF wild-type stage IV vaginal melanoma presented to the medical oncology clinic for a second opinion. The clinical course is summarized in **Figure 1**. She was initially diagnosed with vaginal melanoma 3 years prior after developing post-menopausal vaginal bleeding. Imaging showed a 2.8-cm × 3.6-cm × 3.7-cm vaginal mass with a 1.5-cm perirectal lymph node. Biopsy of the vaginal mass was consistent with melanoma, and staging PET/CT showed no distant metastases. She was started on pembrolizumab and received vaginal radiation in six fractions. After 9 months on pembrolizumab, there was interval development of a biopsy-proven right adrenal metastasis for which she received CyberKnife radiotherapy. Pembrolizumab was continued for an additional 8 months, at which time she developed a cough. CT neck showed mild oropharyngeal enhancing mucosal thickening consistent with pharyngitis and bilateral aryepiglottic fold thickening suspicious for laryngitis. Pembrolizumab was stopped, pantoprazole was initiated, and her symptoms resolved.

Eight months later, while off treatment, she presented to our office with iron deficiency anemia. PET/CT showed jejunal wall thickening concerning for a new site of metastasis, which was confirmed as melanoma by biopsy via push enteroscopy. Surgical resection was considered but declined due to the complexity of the procedure. Dual checkpoint inhibitor therapy with ipilimumab and nivolumab was initiated.

**Figure 2:**
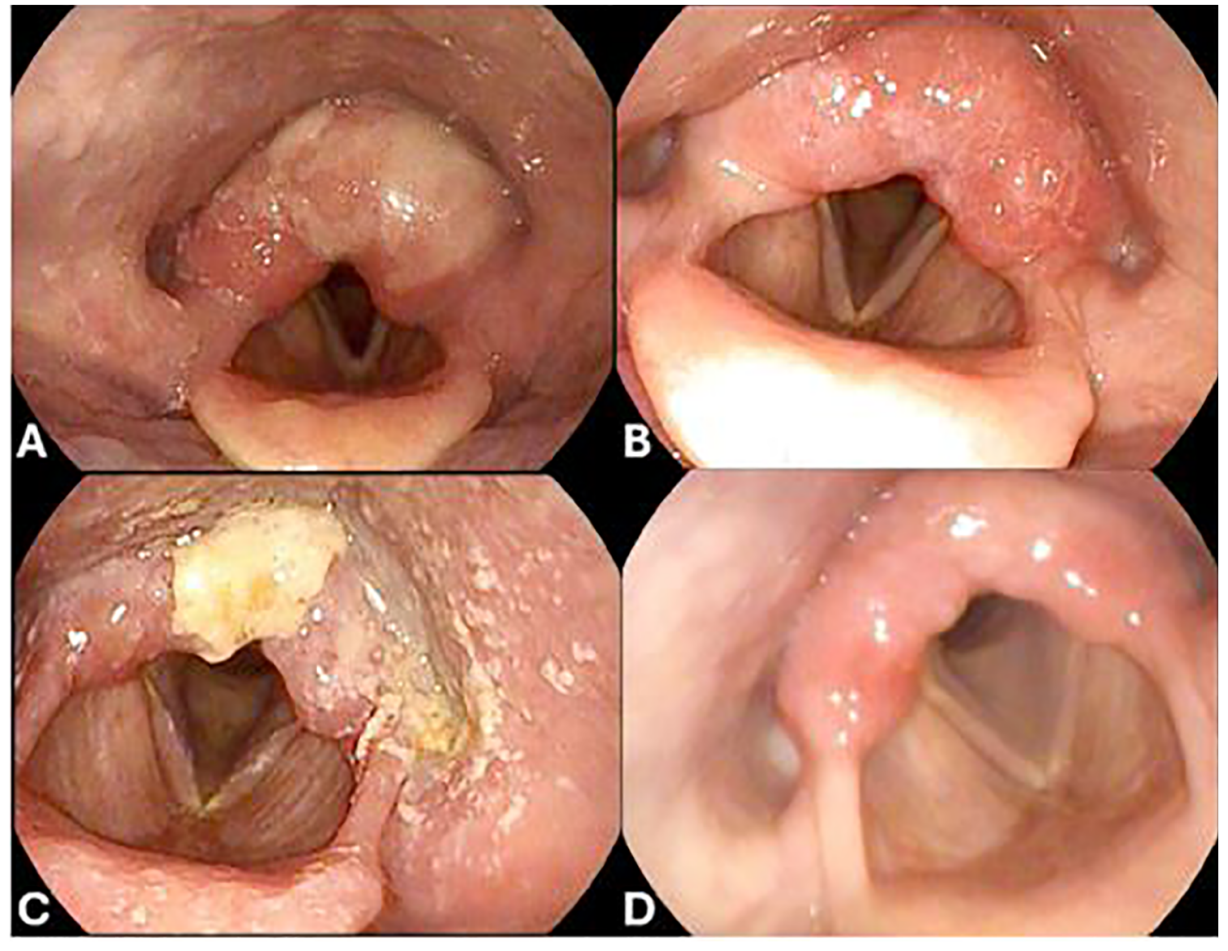
Video laryngoscopy after the third cycle of ipilimumab/nivolumab, which shows moderate edema of the left greater than the right arytenoids with scant superficial exudate **(A)**. Video laryngoscopy after holding dual ICI therapy, 12 days of steroids, and being treated with one dose of intravenous methylprednisolone, which showed improvement of edema, with the left vocal fold and left pyriform clear of edema **(B)**. Video laryngoscopy after retrial of nivolumab, which shows posterior pharyngeal wall fungal elements consistent with thrush, worse supraglottic debris, and mild left glottic edema **(C)**. Video laryngoscopy after completion of the final steroid course, antibiotics, and antifungal agents following nivolumab rechallenge, which showed no evidence of thrush or edema **(D)**.

After three cycles of ipilimumab/nivolumab, the patient presented with worsening cough, left-sided throat pain, dysphonia, and dysphagia to solids and liquids. Video laryngoscopy showed moderate edema of the left greater than the right arytenoids with scant superficial exudate but without airway obstruction [Common Terminology Criteria for Adverse Events (CTCAE) v5 grade 2] (**Figure 2A**). Immunotherapy was held, and she was started on prednisone 40 mg daily and amoxicillin–clavulanate. Due to ongoing edema and unchanged grade 2 symptoms after 1 week, steroids were increased to intravenous methylprednisolone 80 mg once followed by prednisone 80 mg daily. After 5 days, repeat video laryngoscopy showed significant improvement, with the left vocal fold and left pyriform clear of edema (**Figure 2B**), so a steroid taper was initiated over a course of 6 weeks. PET/CT demonstrated a partial response in the small intestine with no new disease.

Following steroid taper and resolution of arytenoid edema, ipilimumab was discontinued and a retrial of nivolumab monotherapy was attempted. However, 1 month later, the cough and dysphonia recurred. Immunotherapy was held and she received prednisone 40 mg daily, doxycycline, and inhaled steroids. Video laryngoscopy showed recurrent edema, more in the left arytenoid and post-cricoid area, without airway obstruction. Given the persistent symptoms, prednisone was increased to 60 mg daily. One week later, video laryngoscopy revealed posterior pharyngeal wall fungal elements consistent with thrush, with right supraglottic debris more than the left and improvement in but still present left glottic edema (**Figure 2C**). A biopsy of the right posterior arytenoid showed detached reactive/inflamed squamous epithelium with fungal organisms, consistent with *Candida*. Nystatin oral suspension, fluconazole, and penicillin V were started. The steroid inhaler was discontinued and a prednisone taper was initiated. Within 1 week, the thrush resolved and the edema significantly improved. Two months from the start of her recurrent symptoms, video laryngoscopy showed no glottic edema (**Figure 2D**).

Immunotherapy remained on hold. The patient declined surgical resection of the residual small bowel metastasis and received focal radiation (3,750 cGy) instead. Two months after radiation therapy, supraglottitis recurred but quickly responded to steroids. Rheumatologic evaluation was conducted to assess the need for chronic immunosuppression, but there were no further recurrences. Nearly 7 months from her last immunotherapy dose, PET/CT continued to show an ongoing response, with no new disease and interval improvement of the small bowel wall metastasis.

## Discussion

This case contributes to the literature on upper airway-associated irAEs, which are rarely reported (**Table 1**). Our patient presented with cough, dysphagia, and globus sensation and was found to have supraglottic edema responsive to corticosteroids, which recurred twice after a rechallenge of nivolumab monotherapy. In retrospect, it is possible that her prior vague cough while on pembrolizumab monotherapy may have been the first low-grade occurrence of supraglottitis.

ICI-related supraglottitis is a diagnosis of exclusion. Infectious and other non-immune causes must be ruled out, especially given the overlapping symptoms. In this case, the diagnosis was supported by symptom resolution with corticosteroids, independent of antibiotic use, and reproducible recurrence upon ICI rechallenge. Although an arytenoid biopsy was performed during a recurrent episode, it only demonstrated *Candida*, due to concurrent thrush at the time. Importantly, multidisciplinary care with regular otolaryngology (ENT) input was critical to safe evaluation and management.

Gascon et al. reported ICI-induced supraglottitis in two patients, one with melanoma on nivolumab and relatlimab and another patient with bladder cancer on pembrolizumab (3). Both patients developed symptoms 4 months after ICI initiation and had severe epiglottic edema requiring a medical intensive care unit stay and intravenous steroids. In contrast, our patient developed symptoms approximately 4 weeks after starting ipilimumab/nivolumab, and her presentation was less severe, responding to a dose of approximately 1 mg/kg of prednisone. Notably, our patient—as well as both cases described by Gason et al.—experienced recurrence of supraglottitis following completion of the initial steroid course, necessitating additional corticosteroid therapy.

Risk factors for ICI-induced supraglottitis remain unclear. Our patient had psoriasis, which mildly flared while on treatment, and although it did not require intervention, her underlying autoimmunity may have increased the risk of irAE development. She was referred to a rheumatologist for further evaluation, and undiagnosed psoriatic arthritis was considered. Supraglottitis has also been reported in autoimmune diseases such as rheumatoid arthritis, lupus, and others; however, the patient had no evidence of these. Pulmonary function tests to evaluate for autoimmune lung disease were normal, and imaging of the hands and feet showed degenerative changes. No serologic testing was performed. A limitation of this case is the lack of collection of inflammatory biomarkers (e.g., CRP, ESR), which may have helped support the immune-mediated nature of these events or aided in monitoring for resolution or recurrence of supraglottitis. Future directions include identifying clinical, immunologic, and genetic risk factors that may predispose patients to airway-associated irAEs. This includes evaluating the role of underlying autoimmune conditions, baseline inflammatory markers, and immune profiling that might predict the development of severe irAEs in general. Ultimately, this would allow clinicians to tailor ICI treatment based on irAE risk, for example, whether to choose ICI monotherapy over combination therapy, or when to consider prophylactic approaches in high-risk patients.

In our patient, rechallenge with nivolumab monotherapy led to recurrent supraglottitis. The decision to resume ICI therapy following a severe irAE remains patient-specific. The risks and benefits must be weighed in the context of disease control, the availability of effective alternative treatments, and the ability to safely manage a recurrent irAE. Many clinical trials mandate permanent discontinuation of treatment after grade 3–4 irAEs, but real-world clinical practice is more variable. According to the 2021 Society for Immunotherapy of Cancer (SITC) clinical practice guidelines, ICI rechallenge may be considered in patients with grade 2 irAEs if symptoms have resolved or are controlled with less than 10 mg of prednisone daily. For patients with prior grade 3 irAEs, the decision to resume therapy should involve a careful assessment of risks and benefits (5). Some experts have proposed the use of prophylactic immunosuppressants during rechallenge to mitigate recurrence, and this approach should be further investigated (6).

In conclusion, airway-associated irAEs are largely underrecognized given their vague symptomatology, such as throat discomfort, dysphagia, and cough, which can often be attributed to alternative etiologies. However, persistent symptoms of cough or globus sensation during ICI therapy should prompt ENT evaluation and visual inspection. Early recognition and multidisciplinary care are essential to mitigate the risk of life-threatening airway obstruction.

## Data Availability

The original contributions presented in the study are included in the article/supplementary material. Further inquiries can be directed to the corresponding author.
